# Supplementary material for: The French Early Breast Cancer Cohort (FRESH): A Resource for Breast Cancer Research and Evaluations of Oncology Practices Based on the French National Healthcare System Database (SNDS)
Source: Cancers (Basel). 2022 May 27;14(11):2671. doi: 10.3390/cancers14112671 (PMC9179405; doi:10.3390/cancers14112671)
Supplement: Supplementary file 1 [file cancers-14-02671-s001.zip › cancers-1677125-supplementary.pdf]

Supplemental material

Patient selection

We applied 10 filters for patient selection (Fig. S1):

- (1) BC diagnosis was identified by a tag with an ICD-10 diagnosis code for BC (Table S1) in long-term illness (LTI) records; or in at least one hospital discharge report within the period considered. The inclusion year was defined as the year of the first diagnosis code for BC.
- (2) Patients were considered to have BC newly diagnosed between January 1, 2011 and December 31, 2017 if the inclusion year was between 2011 and 2017. Patients with a BC diagnosis code in 2010 were excluded from the analyses.
- (3) Female patients were identified from the binary sex variable directly available in ODP. Male patients were excluded from the cohort.
- (4) Age at BC diagnosis was calculated as the rounded difference, in years, between the date of BC diagnosis and the patient's date of birth. Patients under 18 years of age at BC diagnosis or whose date of birth was missing in the ODP were excluded.
- (5) Registration to general health insurance coverage plan ("Régime Général") was identified from the insurance scheme table available in ODP, which summarizes the start and end date of the patient's insurance scheme. Patients affiliated to another insurance scheme or with a change of affiliation between the inclusion year and 2018 were excluded.
- (6) BC surgery was identified as described in the surgery section. Women with no breast surgery at any time in the inclusion year or the following year were excluded from the

study. We defined BC index surgery as the first breast surgery intervention occurring in the inclusion year or the following.

- (7) Patients suspected to have been diagnosed with BC at the same time as cancer at another site were excluded. These patients were identified by at least one diagnosis code (hospital discharge or LTI) for the other cancer (Table S2), except for cervical intraepithelial neoplasia and basal or squamous cell carcinoma of skin, or by the administration of chemotherapy or immunotherapy molecules not indicated for BC (Table S2) in the year preceding or following BC index surgery.
- (8) We excluded diagnoses of BC presenting as locoregional or distant relapses from a previous cancer diagnosed before the study period, by excluding patients with an LTI for BC starting before January 1, 2011, or a diagnosis code for a prior BC (ICD-10 Z853) before or up to six months after BC index surgery.
- (9) Stage IV BC at diagnosis was suspected on the basis of at least one diagnosis code for metastatic disease (Table S3); or at least one hospital administration or outpatient delivery of chemotherapy, targeted therapy or endocrine therapy molecules indicated only for metastatic disease (Table S3); or at least three days of daily chemotherapy sessions before or up to six months after BC index surgery; or more than 20 cycles of anti-*HER2* targeted therapy. The corresponding patients were excluded from the study population. The methodology used to identify daily chemotherapy sessions and the number of cycles of anti-*HER2* targeted therapy is detailed below.
- (10) Data quality was checked to exclude patients with missing data for area of residence, inconsistent numbers of chemotherapy sessions within a hospital stay or a refined date of diagnosis in 2010 or 2018, based on treatment dates and biopsy procedure dates.

BC treatments

Surgery

Starting with the BC index surgery and then following successive hospitalizations, the surgical procedures occurring after the BC index surgery were considered to be revision surgery interventions provided that (i) they occurred within three months of the previous surgical intervention, (ii) they constituted the first surgical procedure after adjuvant chemotherapy, less than three months after the last chemotherapy session. The methodology used to identify adjuvant chemotherapy is detailed below. Final binning for breast surgery into two categories was as follows: (1) *mastectomy,* if at least one operation from among the BC index surgery and revision surgery interventions was mastectomy, with or without axillary surgery; (2) *partial mastectomy* otherwise. Final binning for axillary surgery into two categories was as follows: (1) *Yes*; if at least one of the surgical interventions from among the BC index surgery and the revision surgery operations was mastectomy with axillary surgery, partial mastectomy with axillary surgery or axillary surgery without breast surgery; (2) *No*; otherwise.

Radiotherapy

Radiotherapy (RT) sessions were tagged as neoadjuvant if they occurred before BC index surgery and adjuvant otherwise.

Radiotherapy settings were tagged as: (1) *neoadjuvant* if all RT sessions were tagged as neoadjuvant; (2) *adjuvant* if all RT sessions were tagged as adjuvant; (3) *both* if at least one RT session was tagged as neoadjuvant and at least one RT session was tagged as adjuvant.

The neoadjuvant *RT start date* was defined as the date of the first session of neoadjuvant RT between 150 days before BC index surgery and BC index surgery. The neoadjuvant *RT end date* was defined as the date of the last session of neoadjuvant radiotherapy within 150 days of the neoadjuvant RT start date.

The adjuvant *RT start date* was defined as the date of the first session of adjuvant RT from BC index surgery to 365 days after BC index surgery. The adjuvant *RT end date* was defined as the date of the last session of adjuvant radiotherapy within 150 days of the adjuvant RT start date.

Chemotherapy

Chemotherapy setting and calculation of chemotherapy dates

CT sessions were tagged as neoadjuvant if they occurred before the BC index surgery date and adjuvant otherwise.

CT settings were tagged as: (1) *neoadjuvant* if all CT sessions were tagged as neoadjuvant; (2) *adjuvant* if all CT sessions were tagged as adjuvant; (3) *both* if at least one CT session was tagged as neoadjuvant and at least one CT session was tagged as adjuvant.

The neoadjuvant *CT start date* was defined as the date of the first session of neoadjuvant CT between 250 days before BC index surgery and BC index surgery. The neoadjuvant *CT end date* was defined as the date of the last session of neoadjuvant CT within either seven months of the neoadjuvant CT start date or the first date of suspicion of metastatic progression on treatment (administration of molecules indicated only for metastatic disease listed in Table S3 or at least three consecutive daily chemotherapy sessions), whichever occurred first.

The adjuvant *CT start date* was defined as the date of the first session of adjuvant CT between BC index surgery to 180 days after BC index surgery. The adjuvant *CT end date* was defined as the date of the last session of adjuvant CT within either seven months of the adjuvant CT start date or the first date of suspicion of metastatic progression on treatment (administration of molecules indicated only for metastatic disease listed in Table S3 or at least three consecutive daily chemotherapy sessions), whichever occurred first.

Computation of intervals between chemotherapy sessions

Hospital stays (outpatient care or long-term hospitalizations) including CT sessions were characterized by their start date, end date and the number of CT sessions performed. Patients with any hospital stay with an inconsistent number of CT sessions (*i.e.* more than 1 session per day) were excluded from the cohort. We then distinguished two categories of patients.

- The first category included patients for whom CT sessions were all performed in the day hospital (hospital outpatient care), such that the exact date of each CT session was known. Most of the patients (96.7%) belonged to this category. For these patients, the intervals between two consecutives CT sessions were calculated as the difference in CT session dates, rounded to 1, 7, 14 or 21 days.
- The second category included patients for whom at least one CT session date was unknown, making it impossible to calculate directly all the intervals between consecutive CT sessions. Only a minority of patients belonged to this category (3.3 %). We inferred the sequence of CT session intervals for these patients from the most frequent sequence of intervals for patients in the first category that matched the available data for the timing of CT sessions for the patient concerned. If the inferred sequence of

intervals was not among the 20 most frequent sequences for patients in the first category, the inference was considered uncertain and the patient was excluded from the cohort.

Daily chemotherapy sessions (sessions separated by an interval of one day) were tagged as indicating a suspicion of metastatic disease. If at least three consecutive daily CT sessions were identified within six months of the BC index surgery, the patient was considered to have had stage IV BC at diagnosis and was excluded from the cohort. If at least three consecutive daily CT sessions were identified six months or more after BC index surgery, the patient was assumed to have distant metastases or disease progression, and all subsequent CT sessions were excluded from calculation of the CT regimen.

Identification of CT regimen and the number of cycles

Apart from costly innovative drugs part of a special reimbursement process in France called "list en sus"), chemotherapy molecules were not directly identifiable in hospital care. Nevertheless, chemotherapy regimen may still sometimes be inferred from specific temporal schemes (*e.g.* paclitaxel is the only early BC chemotherapy molecules to be delivered weekly). The decision rules applied to patients without targeted therapies are displayed in Fig. S2:

(1) Any succession of seven-day intervals was considered to identify a paclitaxel regimen (yellow label).

(2) At least three 14-day intervals identified a dose-dense anthracycline regimen: 14-day delays followed by (i) several 21-day intervals were tagged as anthracycline-docetaxel regimens (orange label); (ii) several seven-day intervals were tagged as anthracycline-paclitaxel regimens (red label).

(3) Any 21-day intervals followed by seven-day intervals were inferred to correspond to anthracycline-paclitaxel regimens (red label).

(4) The remaining 21-day intervals could reflect treatment with either anthracycline or docetaxel. Before March 2012, docetaxel was identifiable in the SNDS because it was part of a special reimbursement process in France, called "*list en sus*", for costly and innovative molecules. A 21-day interval before March 2012 not reimbursed as docetaxel was tagged as an anthracycline regimen. Hence, sequences of 21-day intervals before March 2012 were fully identifiable as anthracyclines-docetaxel (orange label), anthracyclines (dark blue label) or docetaxel (light blue label). After March 2012, it was no longer possible to distinguish between anthracyclines and docetaxel: sequences of 21-day intervals were, therefore, tagged as "Unknown" (gray label).

(5) Finally, combinations of 7, 14 and 21-day intervals between CT sessions not classified by the above rules were classified as "other" (green label).

The number of cycles of CT was calculated as the number of anthracycline or docetaxel sessions plus a third of the number of paclitaxel sessions (rounded downwards), and is presented in the third column of Fig. S2.

Endocrine therapy (ET)

We separated ET for cancer treatment from ET for fertility preservation procedures (FPP), by excluding (i) GnRH agonist deliveries during CT (assumed to be prescribed for ovarian protection), (ii) tamoxifen and AI deliveries in the six weeks preceding any embryo or oocyte cryopreservation (assumed to be prescribed for hormonal stimulation, Table S5) and (iii) all GnRH agonist deliveries for patients with less than three deliveries of GnRH agonists in total. A patient was considered to be treated with ET if she had at least one ET delivery between 250 days before to up to 365 days after her BC index surgery, in accordance with clinical practices.

ET start date was defined as the date of the first ET delivery within these dates, and the patient was considered to have been treated with neoadjuvant ET (NET) if ET start date was before the BC index surgery; adjuvant otherwise. We prevented early prescriptions of ET from being considered as NET, by not tagging patients whose NET start date was less than two months from index surgery as receiving NET; and their ET start date was postponed to the date of the first ET delivery after index surgery. No *ET end date* was defined, because ET is a long-term treatment (five to years) and we did not have access to seven years of outpatient care history for all the patients.

In cases of delivery of any ET molecule approved for use in the metastatic setting, such as toremifene, fulvestrant, or formestane (TableS2), the patient was assumed to have distant metastases, and all subsequent ET deliveries were excluded from calculation of the ET regimen.

Targeted therapy (TT)

The *TT start date* was defined as the date of the first session of anti-*HER2* treatment within the dates considered; and patients were considered to have been treated with neoadjuvant targeted therapy (NTT) if the TT start date was before the BC index surgery date; adjuvant otherwise. *Anti-HER2 end date* was defined as the date of the last session before a discontinuation of sessions for at least six months.

In cases of delivery of any TT molecule approved for use in the metastatic setting, such as lapatinib, bevacizumab, everolimus, palbociclib, or alpelisib (Table S2), the patient was assumed to have distant metastases, and *anti-HER2 end date* was considered to be the last date of anti-*HER2* TT before the delivery of these drugs.

Intervals between consecutive TT sessions were rounded to either 7 or 21 days. The *number of cycles of anti-HER2 TT* was calculated as the number of 21-day intervals between sessions, plus a third of the number of sessions separated by intervals of seven days, rounded down.

The diagnostic code does not distinguish between sessions of TT combined with chemotherapy and sessions of TT alone. We inferred that the patients treated with TT also received chemotherapy unless their ET start date was before the second session of targeted therapy, in which case they were said to be treated with "targeted therapy – endocrine therapy (no chemotherapy)". In accordance with standard clinical practices, patients treated with neoadjuvant targeted therapy (NTT) were not assumed to have received adjuvant CT, unless there was evidence of CT use in an adjuvant setting (CT-only sessions or reporting of the use of CT molecules from the "list en sus"). Chemotherapy regimens, the number of cycles of CT, and targeted therapy + chemotherapy regimens were inferred with a specific algorithm detailed in Fig. S3.

CT/TT sessions were classified as chemotherapy-only sessions (CT sessions without TT; gray pictogram) and targeted therapy +/- chemotherapy sessions (denoted as TT+/-CT; orange pictogram), defined as TT sessions that may or may not have included CT. Hospital stays (day hospital or long-term hospitalizations) containing CT/TT sessions were characterized by their start date, end date and the number of sessions. Patients with any hospital stay for which the number of sessions was inconsistent (*i.e.* more than one session per day) were excluded from the cohort. We then distinguished two categories of patients:

- Patients whose CT/TT sessions all took place at the day hospital, such that the exact date of each CT/TT session was known. This category included most of the patients. Intervals between consecutive CT/TT sessions were calculated as the difference in CT/TT session dates, rounded to 1, 7, 14 or 21 days.
- Patients for whom at least one CT/TT session date was unknown, making it impossible to calculate all the intervals between consecutive 2 CT/TT sessions directly. This category included a minority of patients. We inferred the sequence of CT/TT intervals for these patients from the most frequent sequence of intervals for patients in the first category matching the available data concerning intervals between CT/TT sessions for the patient. If the inferred sequence of intervals was not among the 50 most frequent sequences of intervals for patients in the first category; the inference was tagged as uncertain; and the patient was excluded from the cohort.

Daily chemotherapy sessions (defined as a one-day interval between consecutive sessions) were tagged as indicating a suspicion of metastatic disease. If at least three consecutive daily CT sessions were identified within six months of BC index surgery, the patient was considered to have had stage IV BC at diagnosis and was excluded from the cohort. If at least three consecutive daily CT sessions were identified six months or more after BC index surgery, the patient was assumed to have distant metastases or disease progression, and all subsequent CT sessions were excluded from the calculation of CT regimen.

TT + chemotherapy regimens were then inferred from the sequence of intervals between CT/TT sessions, and the type of session (CT only or TT+/-CT), as detailed in the second column of the Fig. S3. Chemotherapy regimens were directly inferred from TT + chemotherapy regimens (third column of Fig. S3).

The number of cycles of CT was inferred from the number of 7-day, 14-day and 21-day intervals between sessions, as detailed in the fourth column of Fig. S3. This number may not be known, in some cases, due to the impossibility of distinguishing between TT-only and TT+CT sessions. The number of cycles of TT could always be calculated, as the number of 21-day intervals between TT+/-CT sessions plus a third of the number of seven-day intervals between TT+/-CT sessions (rounded down), as detailed in the fifth column of Fig. S3.

Supplemental figures

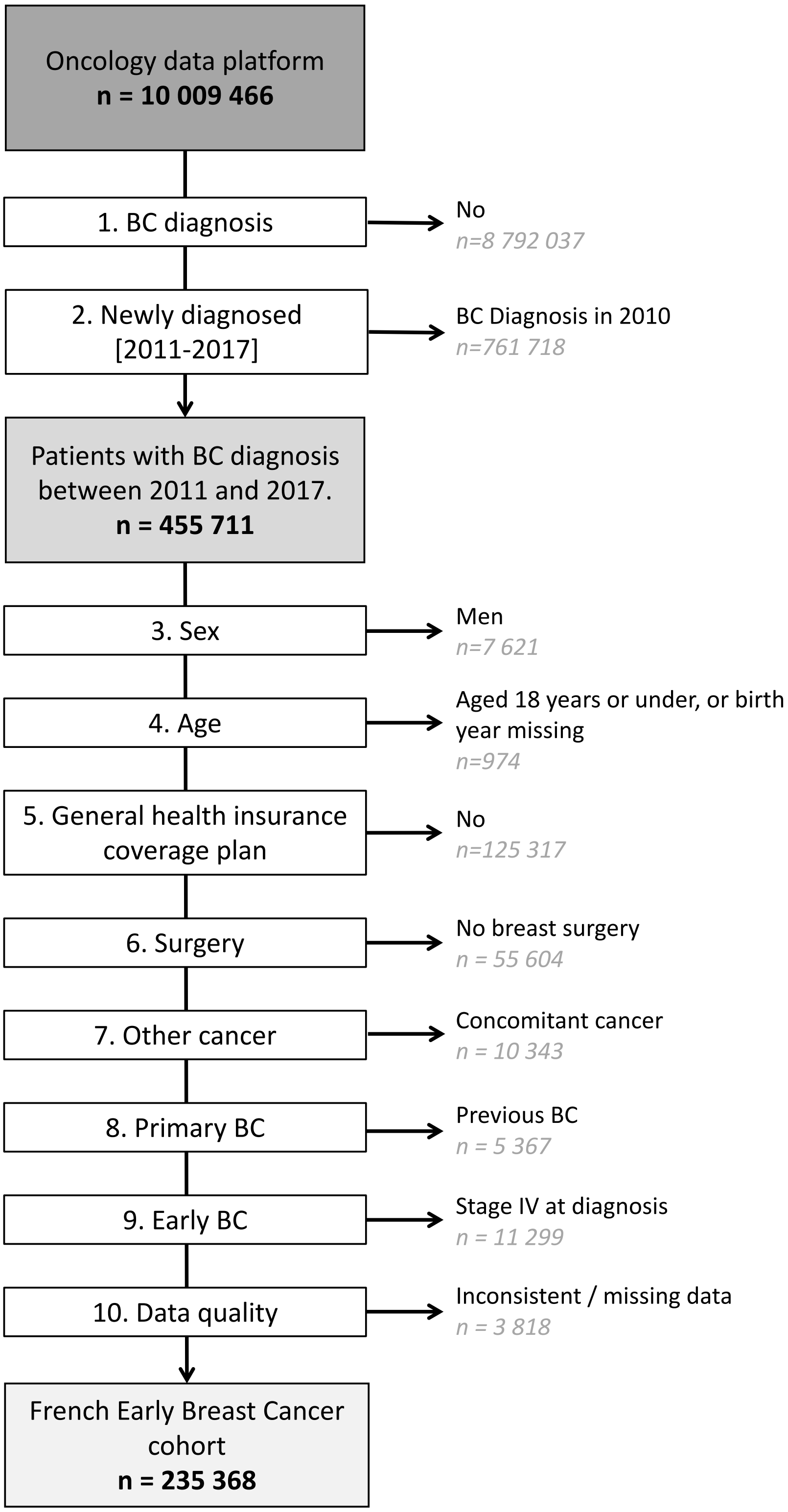

Figure S1: Flowchart of the French Early Breast Cancer Cohort. Abbreviations: BC = breast cancer.

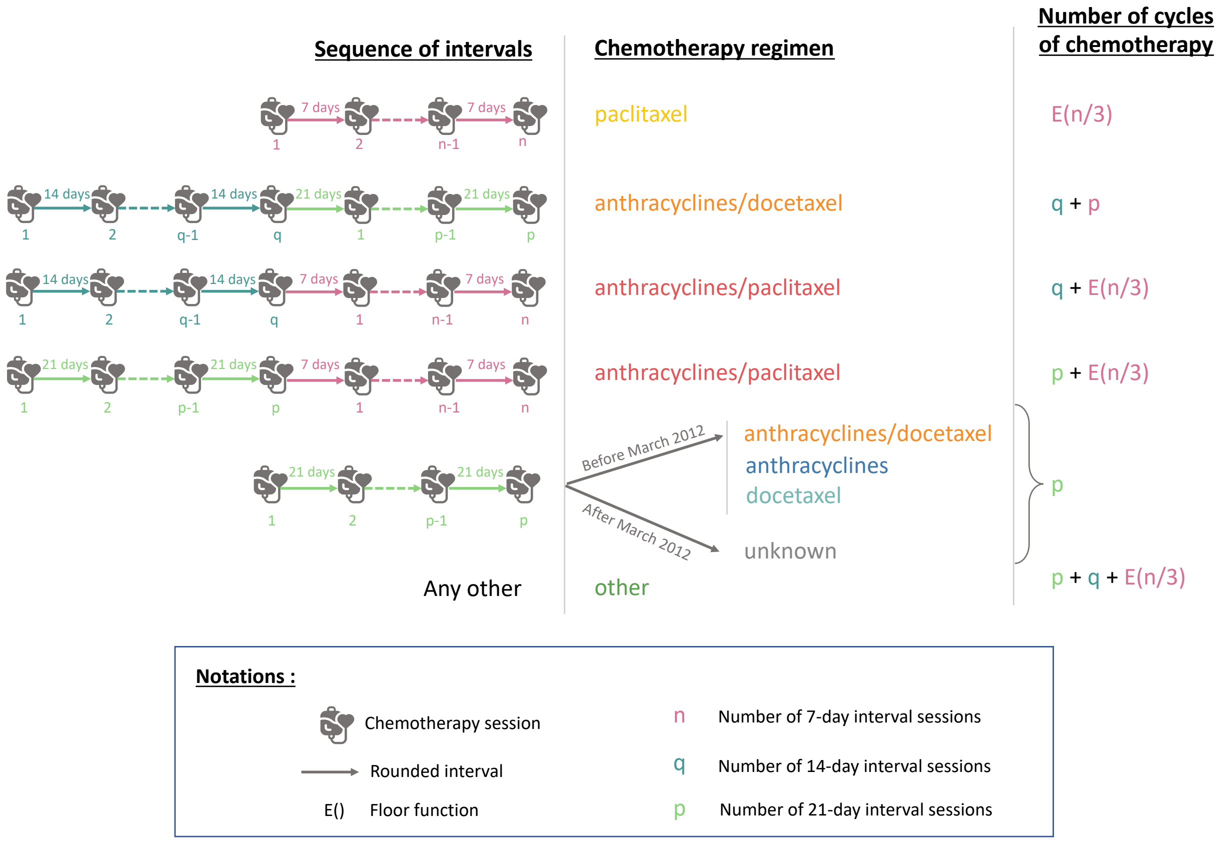

Figure S2: **Rules for the identification of chemotherapy regimens and the number of cycles of chemotherapy in patients not treated with targeted therapy.** The detailed methodology for identifying chemotherapy regimens is described in the Supplementary Material. *n* is the number of seven-day intervals between chemotherapy sessions; *q* is the number of 14-day intervals between chemotherapy sessions; *p* is the number of 21-day intervals between chemotherapy sessions; and E() is the floor function.

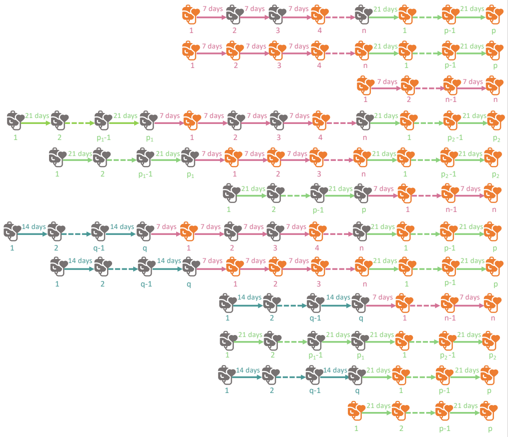

Sequence of intervals	Targeted therapy + Chemotherapy regimen	Chemotherapy regimen	Number of cycles of chemotherapy	Number of cycles of targeted therapy
	paclitaxel-TT (Tolaney)	paclitaxel	E(n/3)	E(n/3) + p
	paclitaxel-TT (Tolaney)	paclitaxel	E(n/3)	E(n/3) + p
	paclitaxel-TT (Tolaney)	paclitaxel	Unknown	E(n/3)
	anthracyclines/paclitaxel-TT	anthracyclines/paclitaxel	p_1 + E(n/3)	E(n/3) + p_2
	anthracyclines/paclitaxel-TT	anthracyclines/paclitaxel	p_1 + E(n/3)	E(n/3) + p_2
	anthracyclines/paclitaxel-TT	anthracyclines/paclitaxel	Unknown	E(n/3) + p
	anthracyclines/docetaxel-TT	anthracyclines/docetaxel	q + E(n/3)	E(n/3) + p
	anthracyclines/docetaxel-TT	anthracyclines/docetaxel	q + E(n/3)	E(n/3) + p
	anthracyclines/docetaxel-TT	anthracyclines/docetaxel	Unknown	E(n/3)
	anthracyclines/docetaxel-TT	anthracyclines/docetaxel	Unknown	p_2
	anthracyclines/docetaxel-TT	anthracyclines/docetaxel	Unknown	p
	docetaxel-TT	docetaxel	Unknown	p
Any other	other	other	Unknown	E(n/3) + p where n is the number of 7-day interval Targeted therapy +/- chemotherapy session and p is the number of 21-day interval Targeted therapy +/- chemotherapy session

Notations :

- Chemotherapy only session
- Targeted therapy +/- chemotherapy session
- Rounded interval
- n Number of 7-day interval sessions
- q Number of 14-day delay sessions
- p Number of 21-day interval sessions

Figure S3: **Rules for the identification of chemotherapy regimens, combinations of targeted therapy and systemic treatment regimens, numbers of cycles of chemotherapy and numbers of cycles of targeted therapy in patients treated with both chemotherapy and targeted therapy.** The detailed methodology for identifying chemotherapy regimens is described in the Supplementary Material. *n* is the number of seven-day intervals between chemotherapy sessions; *q* is the number of 14-day intervals between chemotherapy sessions; *p* is the number of 21-day intervals between chemotherapy sessions; and E() is the floor function.

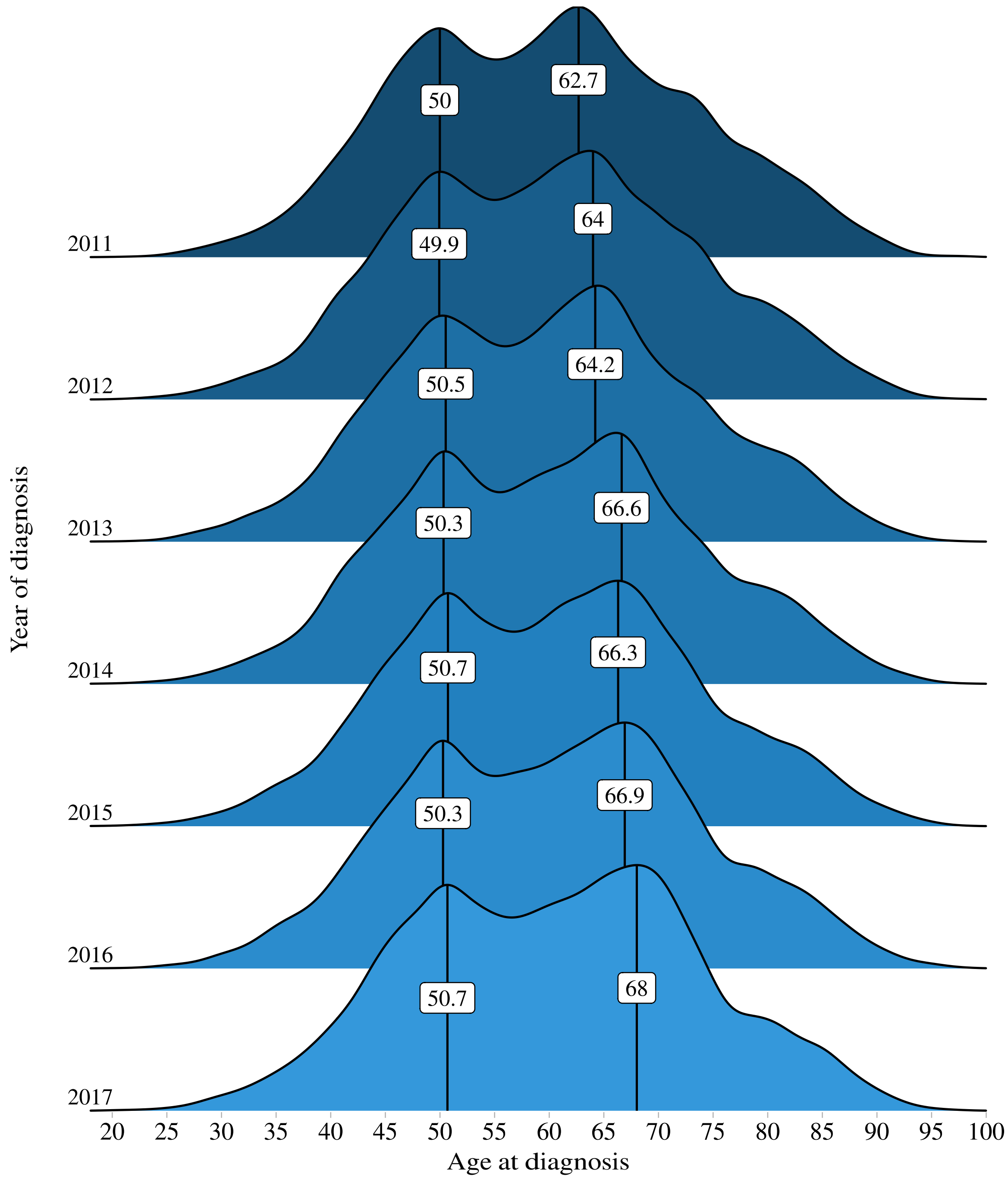

Figure S4: Age at BC diagnosis, by year of BC diagnosis. Distribution of age at diagnosis by year of diagnosis. The hypothesis of unimodality was significantly rejected for all years of diagnosis (p<0.001) and we determined visually that each distribution had two modes. The inferred modes are displayed on the graph.

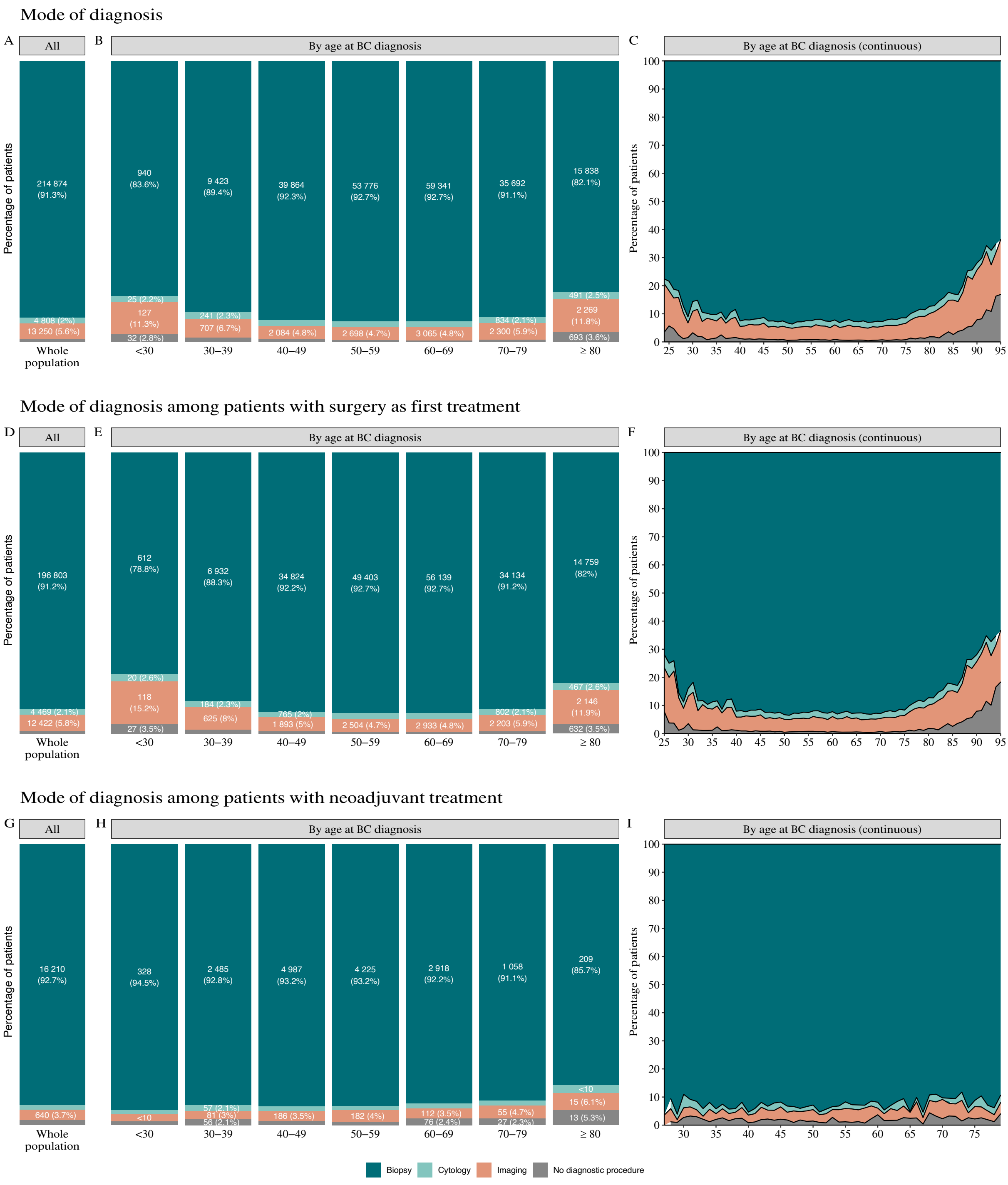

Figure S5: Mode of diagnosis, by age at BC diagnosis

(A) Modes of diagnosis for the total population (*n*=235,368). Raw data for subgroups representing less than 2% of the whole population are not displayed on the graph, to ensure readability: there were 2,436 (1%) patients in the no diagnostic procedure group;

(B) Mode of diagnosis by age class at BC diagnosis. Raw data for subgroups representing less than 2% of the corresponding age class are not displayed on the graph, to ensure readability. The values per age class not displayed are: 30-39 years old: *n*=168 (1.6%), 40-49 years old: *n*=401 (0.9%), 50-59 years old: *n*=408 (0.7%), 60-69 years old: *n*=397 (0.6%), 70-79 years old:

n=337 (0.9%) for the no diagnostic procedure group. The values by age group for the cytology group are: 40-49 years old: *n*=857 (2%), 50-59 years old: 1,121 (1.9%), 60-69 years old: *n*= 1,239 (1.9%);
(C) Mode of diagnosis by age at BC diagnosis. The cohort is restricted to patients aged from 24 to 95 years (*n*=235,152);
(D) Modes of diagnosis in patients with surgery as the first treatment, for the total population (*n*=215,701). Raw data for subgroups representing less than 2% of the whole population are not displayed on the graph, to ensure readability: 2,007 (0.9%) patients had no diagnostic procedure;
(E) Modes of diagnosis for patients with surgery as the first treatment, by age class at BC diagnosis. Raw data for subgroups representing less than 2% of the corresponding age class are not displayed on the graph, to ensure readability. The values by age class not shown are: 30-39 years old: *n*=109 (1.4%), 40-49 years old: *n*=308 (0.8%), 50-59 years old: *n*=339 (0.6%), 60-69 years old: *n*=305 (0.5%), 70-79 years old: *n*=287 (0.8%) for the no diagnostic procedure group. The values by age class for the cytology group are: 50-59 years old: 1,054 (2.0%), 60-69 years old: *n*=1,177 (1.9%);
(F) Modes of diagnosis for patients with surgery as the first treatment, by age at BC diagnosis. The cohort is restricted to patients aged from 25 to 95 years (*n*=215,464);
(G) Modes of diagnosis in patients with neoadjuvant treatment in the total population (*n*=17,479). Raw data for subgroups representing less than 2% of the total population are not displayed on the graph, to ensure readability: there were 323 (1.8%) patients in the no diagnostic procedure group, and 306 (1.8%) in the cytology group;
(H) Modes of diagnosis in patients with neoadjuvant treatment by age class at BC diagnosis. Raw data for subgroups representing less than 2% of the corresponding age class are not displayed on the graph, to ensure readability. The values by age class not shown are: <30 years old: <10, 40-49 years old: 85 (1.6%), 50-59 years old: 61 (1.3%) for the no diagnostic procedure group. The values by age class for the cytology group are: < 30 years old: *n* < 10, 40-49 years old: *n*=90 (1.7%), 50-59 years old: *n*=67 (1.5%), 60-69 years old: *n*=59 (1.9%), 70-79 years old: *n*=21 (1.8%);
(I) Modes of diagnosis in patients with neoadjuvant treatment, by age at BC diagnosis. The cohort is restricted to patients aged from 27 to 79 years (*n*=17,132).
Abbreviations: BC = breast cancer.

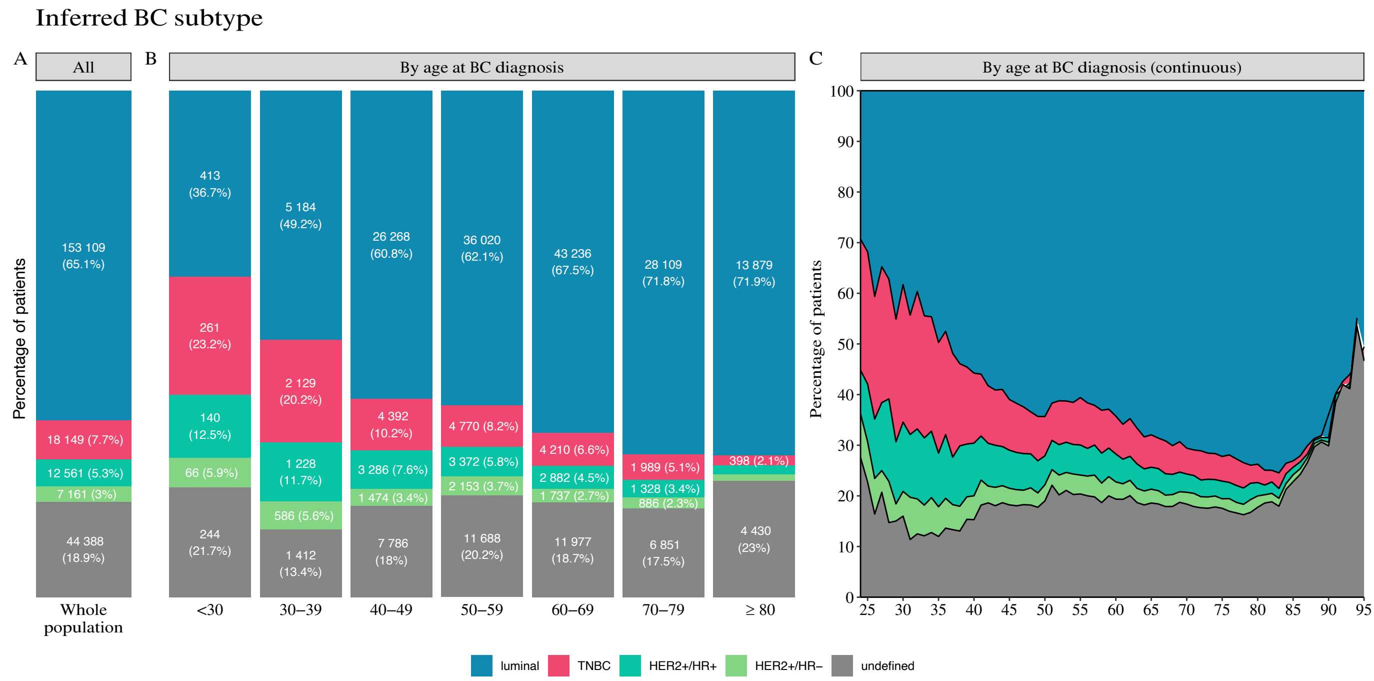

Figure S6: Inferred BC subtype, including undefined tumors, by age at BC diagnosis

(A) Inferred BC subtype for the total population (*n*=235,268);
(B) Inferred BC subtype by age class at BC diagnosis; raw data for subgroups representing less than 2% of the corresponding age class are not displayed on the graph, to ensure readability: ≥ 80 years old: 325 (1.7%) patients in the *HER2*$^{+}$/HR^{+} group and 259 (1.3%) patients in the *HER2*$^{+}$/HR^{-} group.
(C) Inferred BC subtype by age at BC diagnosis. The cohort is restricted to patients aged from 24 to 95 years with an undefined subtype (*n*=235,152).
Abbreviations: BC = breast cancer; HR^{+} = hormone receptor-positive; HR^{-} = hormone receptor-negative; TNBC = triple-negative breast cancer subtype; *HER2*$^{+}$ = human epidermal growth factor receptor 2.

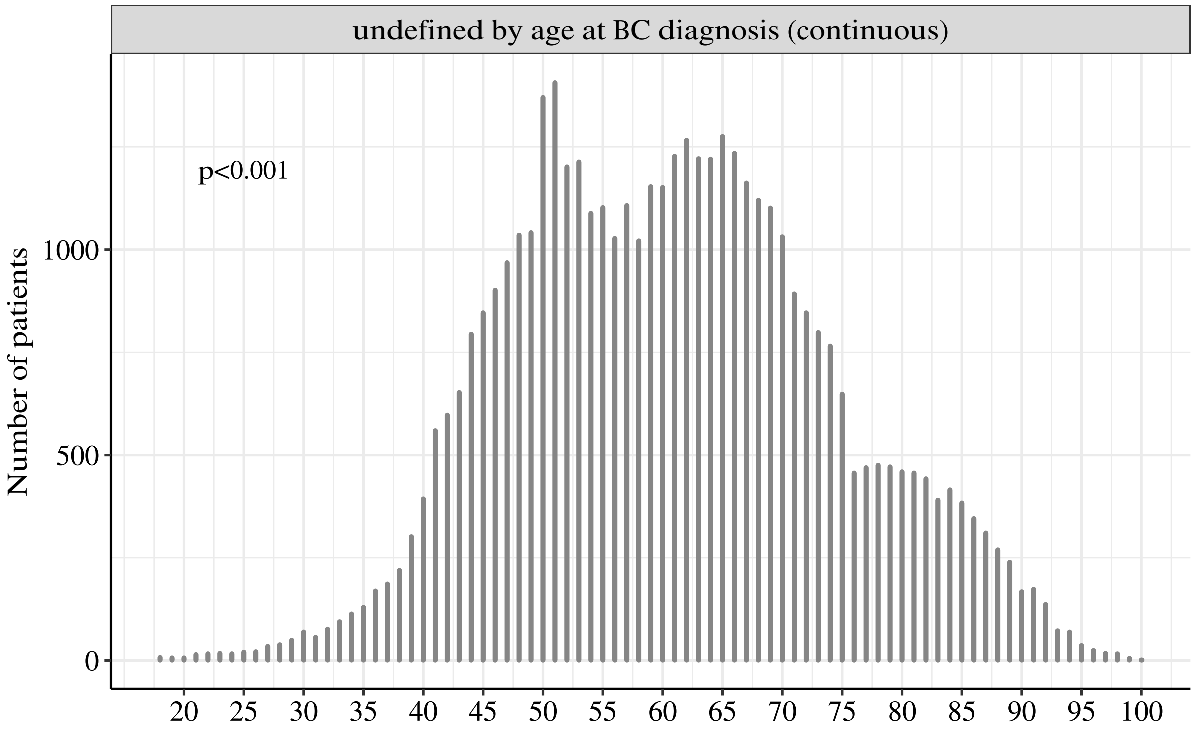

Figure S7: Age at BC diagnosis, for undefined tumors

Age distribution of patients with undefined, inferred subtypes (*n*=44,388) at BC diagnosis (*p*-value for non-unimodality < 0.001).
Abbreviations: BC = breast cancer.

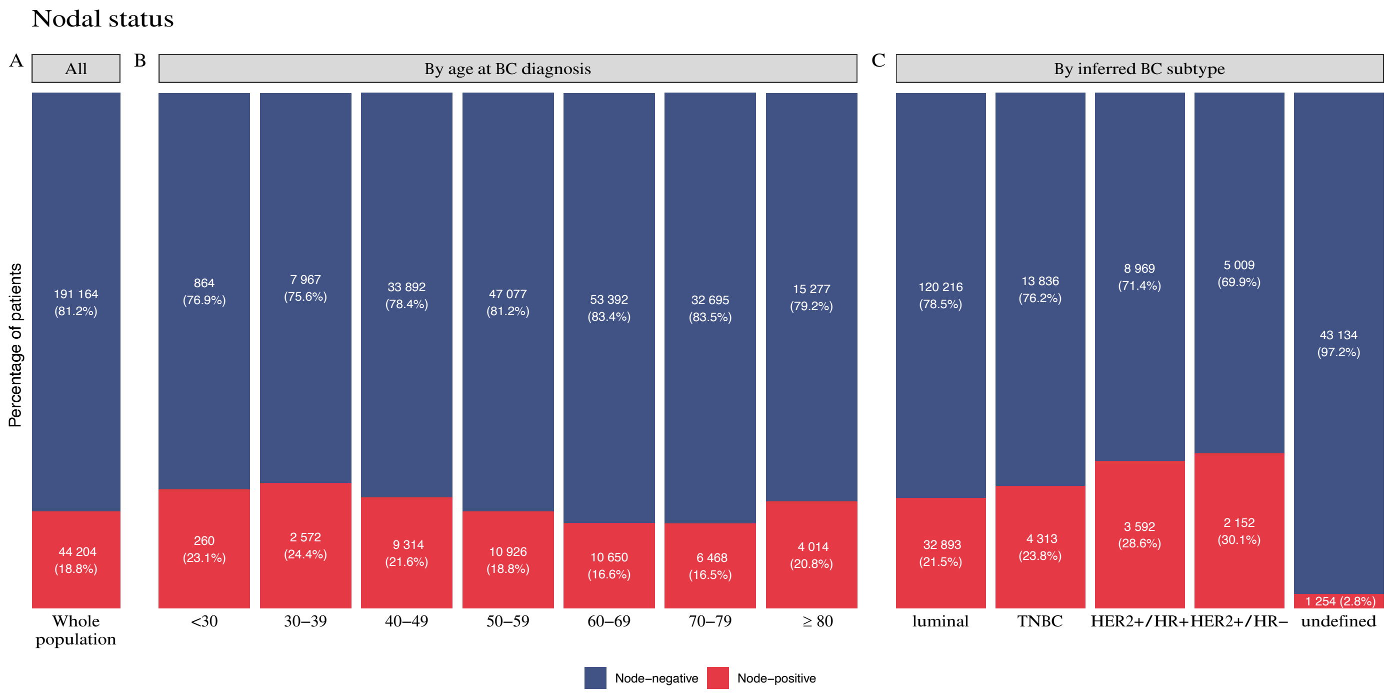

Figure S8: **Nodal status by age at BC diagnosis and inferred BC subtype**

(A) Nodal status in the total population (*n*=235,368);
(B) Nodal status per age class at BC diagnosis;
(C) Nodal status per inferred BC subtype.

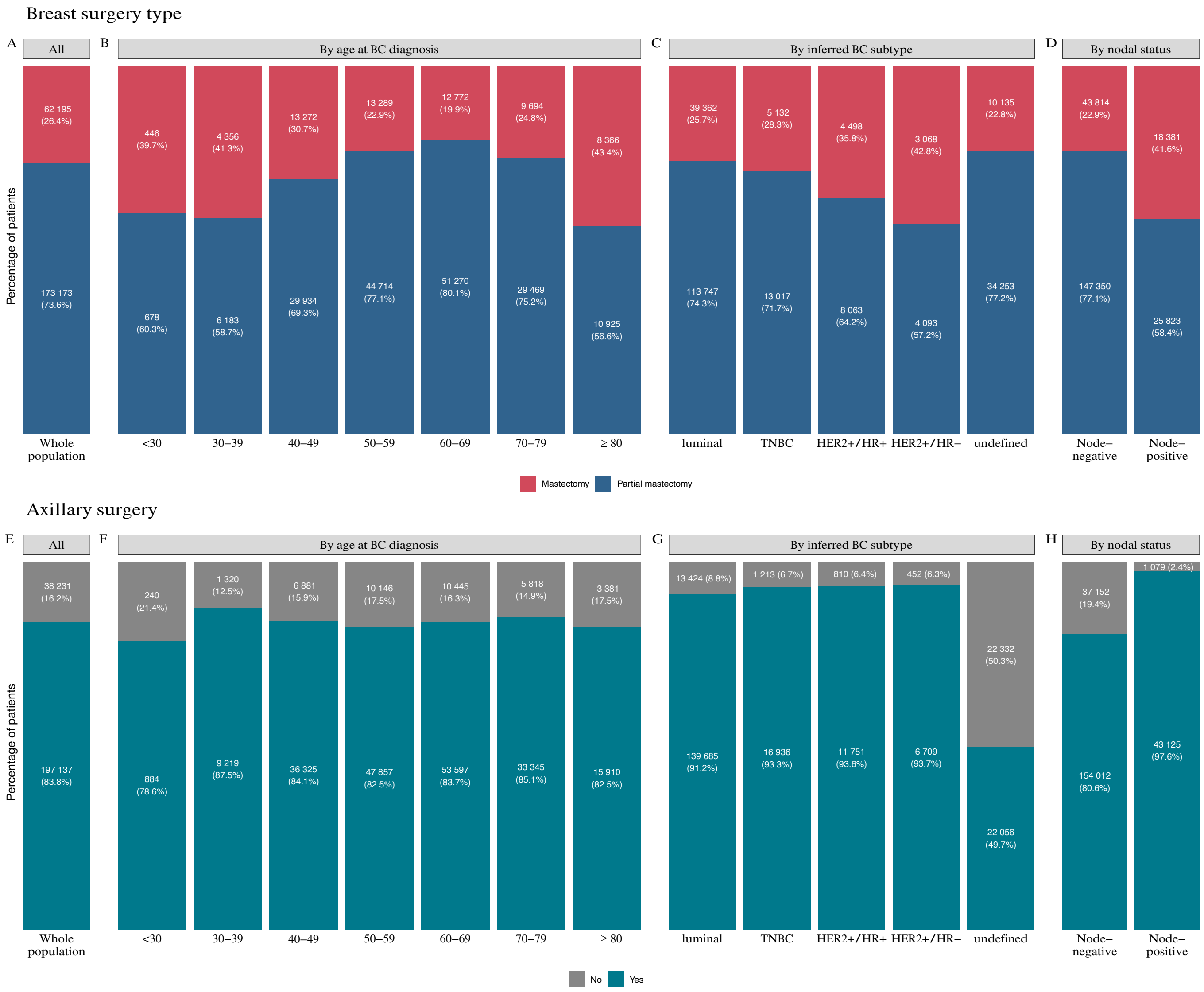

Figure S9: Type of surgery, by age at BC diagnosis, inferred BC subtype and nodal status.

(A) Breast surgery type in the total population (*n*=235,368);
(B) Breast surgery type by age class at BC diagnosis;
(C) Breast surgery type by inferred BC subtype;
(D) Breast surgery type by nodal status at diagnosis;
(E) Use of axillary surgery in the total population (*n*=235,368);
(F) Use of axillary surgery by age class at BC diagnosis;
(G) Use of axillary surgery by inferred BC subtype;
(H) Use of axillary surgery by nodal status at diagnosis.
Abbreviations: BC = breast cancer; TNBC = triple-negative breast cancer subtype; *HER2*$^{+}$ = human epidermal growth factor receptor 2; HR^{+} = hormone receptor-positive; HR^{-} = hormone receptor-negative.

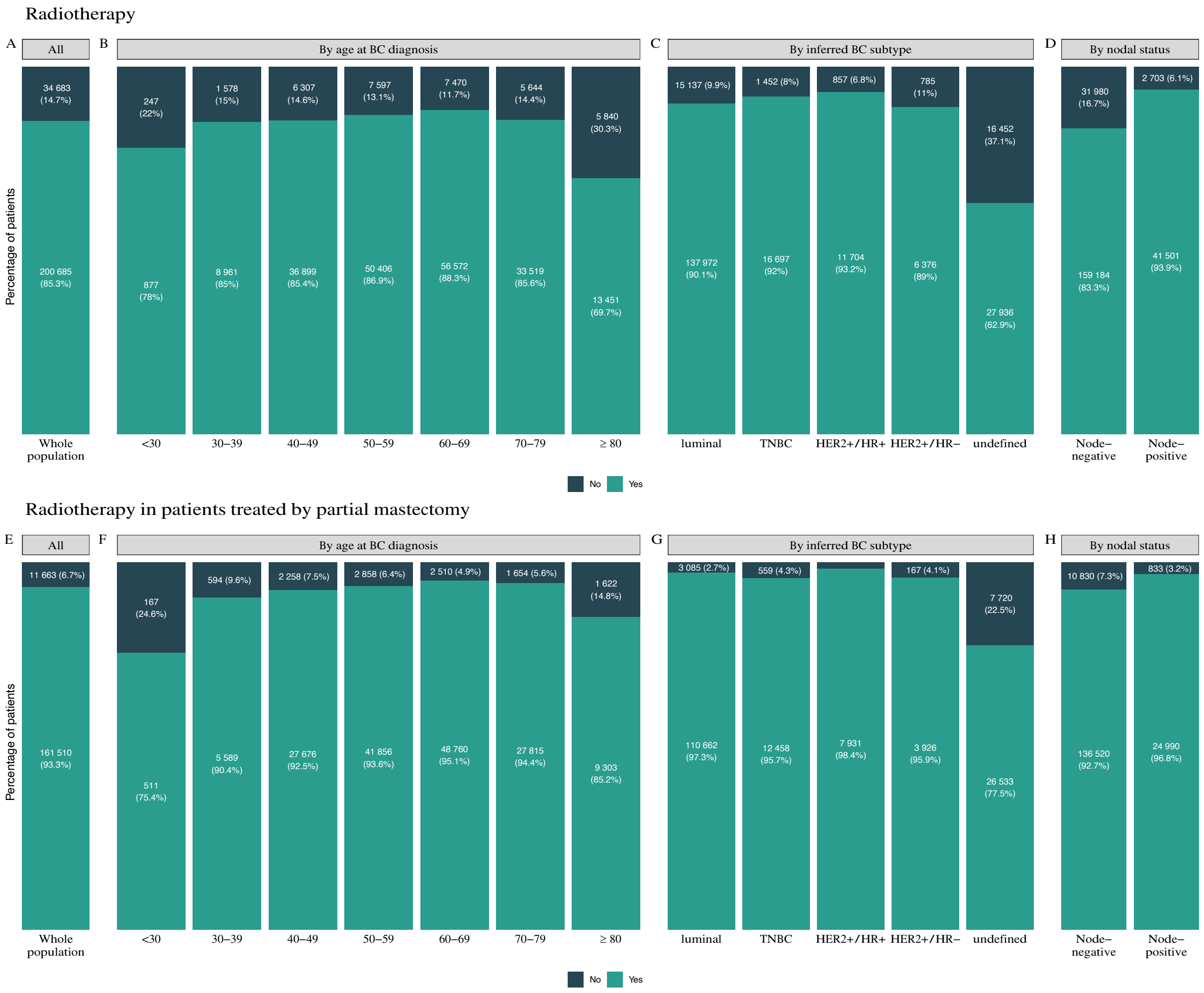

Figure S10: Radiotherapy by age at BC diagnosis, inferred BC subtype and nodal status.

(A) Radiotherapy in the total population (*n*=235,368);
(B) Radiotherapy by age class at BC diagnosis;
(C) Radiotherapy by inferred BC subtype;
(D)Radiotherapy by nodal status at diagnosis;
(E) Radiotherapy in patients treated by partial mastectomy (*n*=173,173);
(F) Radiotherapy in patients treated by partial mastectomy, by age class at BC diagnosis;
(G) Radiotherapy in patients treated by partial mastectomy, by inferred BC subtype. Raw data for subgroups representing less than 2% of the corresponding class are not displayed on the graph, to ensure readability: in the $HER2^{+}$/HR^{+} category, 132 (1.6%) patients did not receive radiotherapy.
(G) Radiotherapy in patients treated by partial mastectomy, by nodal status at diagnosis.
Abbreviations: BC = breast cancer; HR^{+} = hormone receptor-positive; HR^{-} = hormone receptor-negative; $HER2^{+}$ = human epidermal growth factor receptor 2; TNBC = triple-negative breast cancer subtype.

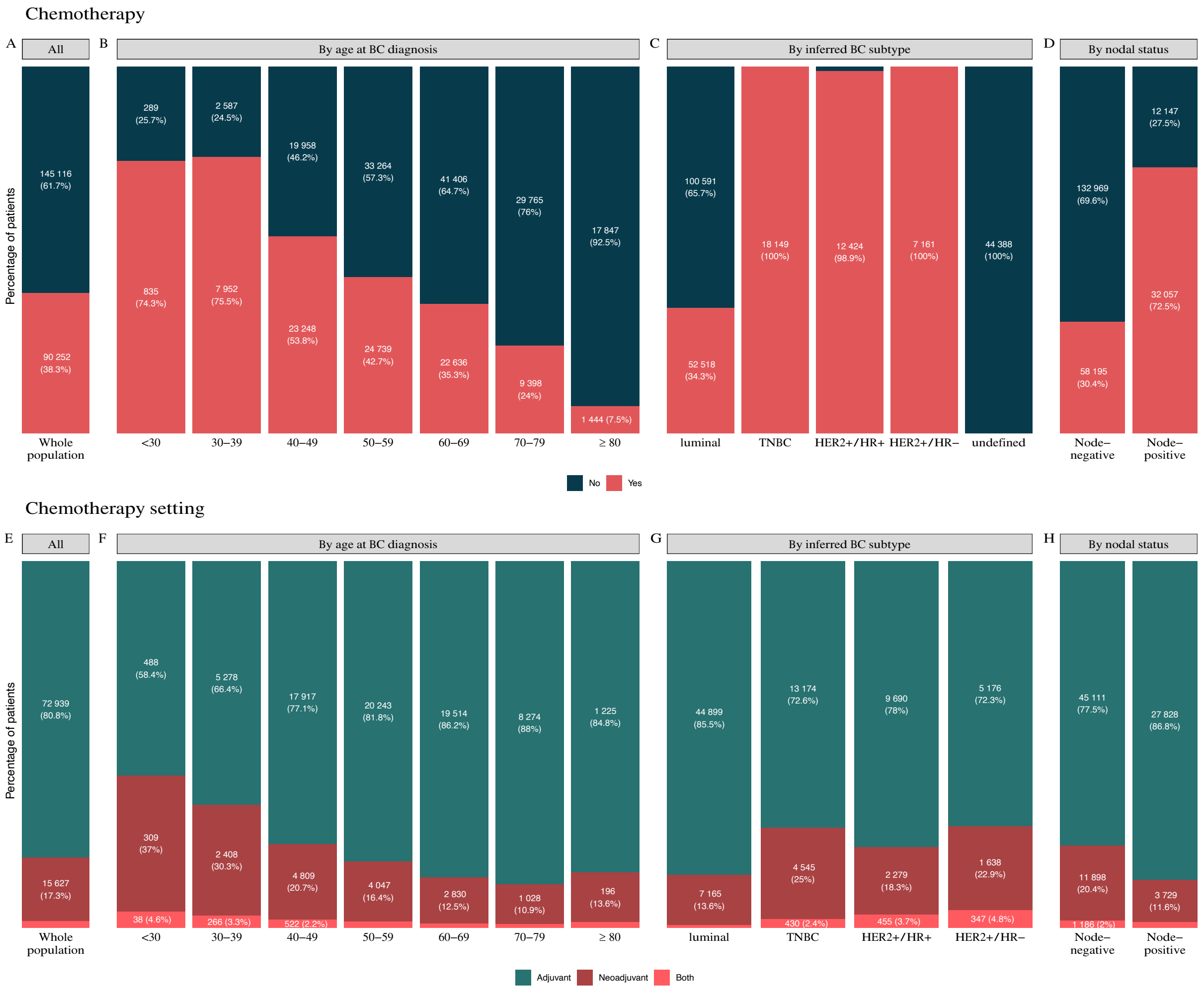

Figure S11: Chemotherapy use and setting by age at BC diagnosis, inferred BC subtype and nodal status

(A) Use of chemotherapy in the total population (*n*=235,368);
(B) Use of chemotherapy per age class at BC diagnosis;
(C) Use of chemotherapy per inferred BC subtype; Raw data for subgroups representing less than 2% of the corresponding breast subtype class are not displayed on the graph, to ensure readability: in the *HER2*$^{+}$/HR^{+} category, there were 137 (1.1%) patients in the no chemotherapy group;
(D) Use of chemotherapy by nodal status;
(E) Chemotherapy setting in the total population (*n*=90,252). Raw data for subgroups representing less than 2% of the total population are not displayed on the graph, to ensure readability: there were 1,686 (1.9%) patients in the "both" category;
(F) Chemotherapy setting by age class at BC diagnosis. Raw data for subgroups representing less than 2% of the corresponding age class are not displayed on the graph, to ensure readability. The values by age class are: 50-59 years old: *n*=449 (1.8%), 60-69 years old: *n*=292 (1.3%), 70-79 years old: *n*=96 (1.0%), ≥ 80 years old: *n*=23 (1.6%) for the "both" group;
(G) Chemotherapy setting by inferred BC subtype; raw data for subgroups representing less than 2% of the corresponding breast subtype class are not displayed on the graph, to ensure readability: in the luminal category, there were 454 (0.9%) patients in the "both" group;

(H) Chemotherapy setting by nodal status; Raw data for subgroups representing less than 2% of the corresponding nodal status group are not displayed on the graph, to ensure readability: in the node-positive category, there were 500 (1.6%) patients in the "both" group;

Abbreviations: BC = breast cancer, HR^{+} = hormone receptor-positive; HR^{-} = hormone receptor-negative; *HER2*$^{+}$=human epidermal growth factor receptor 2; TNBC = triple-negative breast cancer subtype.

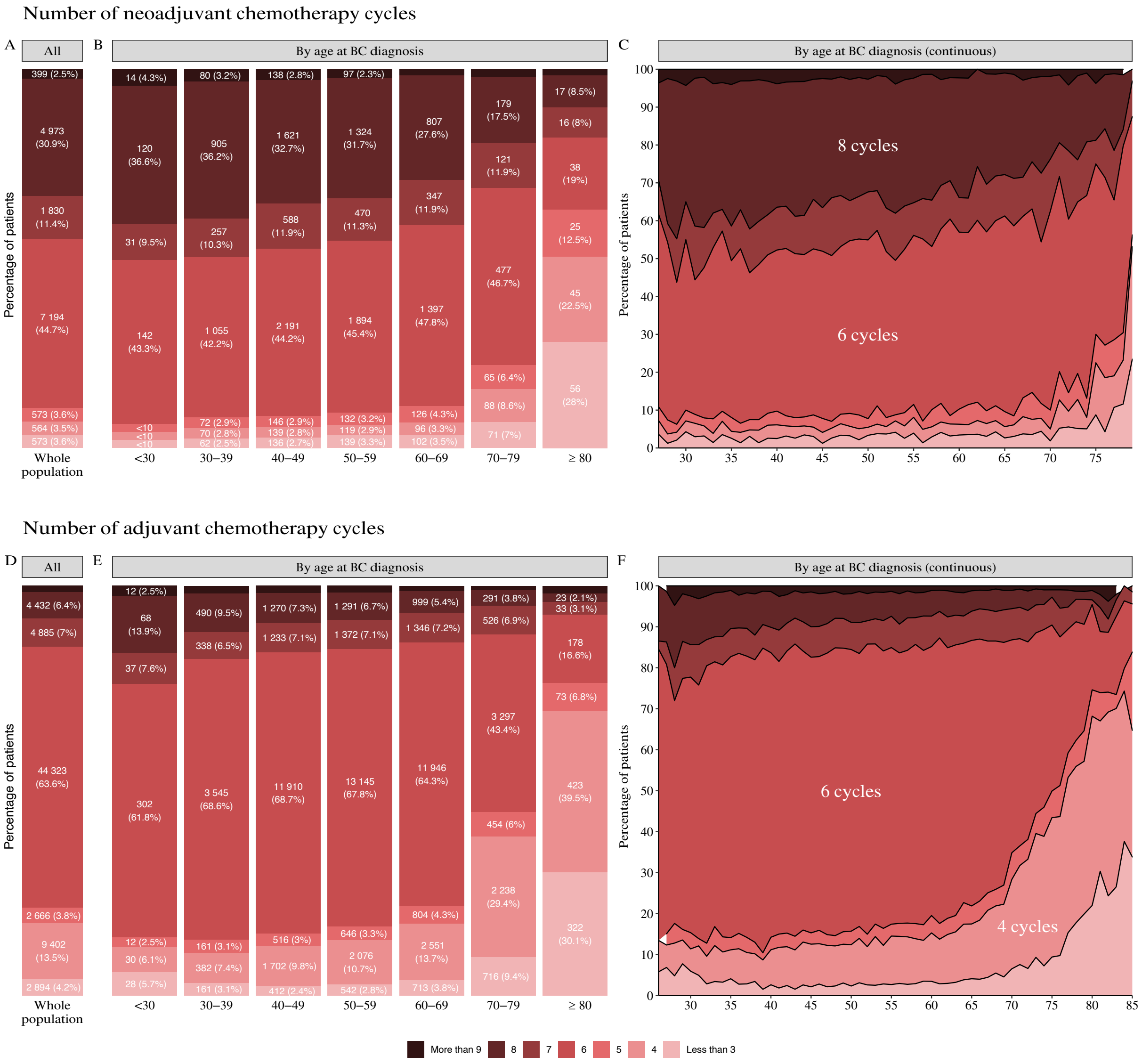

Figure S12: Number of chemotherapy cycles by age at BC diagnosis in the neoadjuvant and adjuvant settings

(A) Number of neoadjuvant chemotherapy cycles for the total population. The number of neoadjuvant chemotherapy cycles was calculated by setting (*n*=16,106). Settings with missing numbers of chemotherapy cycles are not displayed (*n*=1,207). A patient with six

cycles of neoadjuvant chemotherapy followed by four cycles of adjuvant chemotherapy was classified as having six cycles in subfigures A-B-C; and four in subfigures D-E.-F;
(B) Number of neoadjuvant chemotherapy cycles by age class (*n*=16,106) at BC diagnosis. Raw data for subgroups representing less than 2% of the corresponding age class are not displayed on the graph, to ensure readability. The values by age class are: 60-69 years old: *n*=47 (1.6%), 70-79 years old: *n*=20 (2.0%), ≥ 80 years old: *n*<10 for the more than 9 cycles group;
(C) Number of neoadjuvant chemotherapy cycles by age at BC diagnosis. The cohort is restricted to patients aged from 27 to 79 years (*n*=15,810);
(D) Number of adjuvant chemotherapy cycles for the total population (*n*= 69,646). Settings with missing numbers of chemotherapy cycles are not displayed (*n*=4,979). Raw data for subgroups representing less than 2% of the total population are not displayed on the graph, to ensure readability: there were 1,044 (1.5%) patients in the more than 9 cycles group;
(E) Number of adjuvant chemotherapy cycles by age class (*n*=69,646) at BC diagnosis. Raw data for subgroups representing less than 2% of the corresponding age class are not displayed on the graph, to ensure readability. The values by age class are: 30-39 years old: *n*=93 (1.8%), 40-49 years old: *n*=303 (1.7%), 50-59 years old: *n*=315 (1.6%), 60-69 years old: *n*=224 (1.2%), 70-79 years old: *n*=78 (1.0%), ≥ 80 years old: *n*=19 (1.8%) for the more than 9 cycles group;
(F) Number of adjuvant chemotherapy cycles by age at BC diagnosis. The cohort is restricted to patients aged from 26 to 85 years (*n*=69,452).
Abbreviations: BC = breast cancer, More than 9 = more than 9 cycles, 8 = 8 cycles, 7 = 7 cycles, 6 = 6 cycles, 5 = 5 cycles, 4 = 4 cycles, Less than 3 = less than 3 cycles.

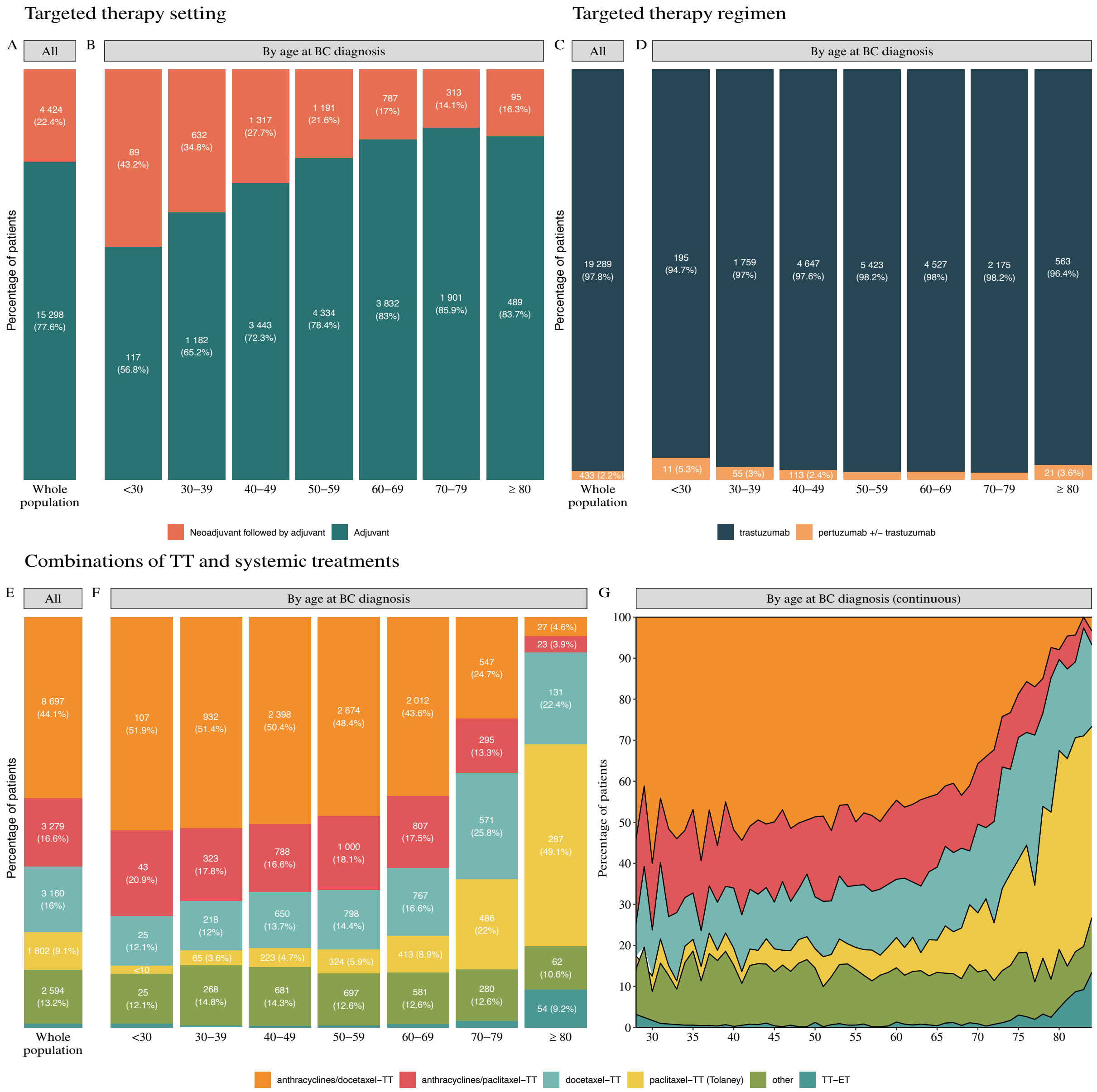

Figure S13: Targeted therapy setting and regimen by age at BC diagnosis

(A) Targeted therapy settings for the total population (*n*=19,722);
(B) Targeted therapy setting by age class at BC diagnosis;
(C) Targeted therapy regimens for the total population (*n*=19,722);
(D) Targeted therapy regimen by age class at BC diagnosis. There were 433 (2.2%) patients in the pertuzumab +/- trastuzumab category, three of whom received pertuzumab only, the remaining 430 being treated with both pertuzumab and trastuzumab. Raw data for subgroups representing less than 2% of the corresponding age class are not displayed on the graph, to ensure readability. The values by age class are: 50-59 years old: *n*=102 (1.8%), 60-69 years old: *n*=92 (2.0%), 70-79 years old: *n*= 39 (1.8%) for the pertuzumab +/- trastuzumab group;

(E) Combinations of TT and systemic treatment regimens for the total population (*n*=19,722). There were 190 (1.0%) patients in the targeted therapy-endocrine therapy group;
(F) Combination of TT and systemic treatment regimen by age class at BC diagnosis. Raw data for subgroups representing less than 2% of the corresponding age class are not displayed on the graph, to ensure readability. The values by age class are: < 30 years old: *n*<10, 30-39 years old: *n*<10, 40-49 years old: *n*=20 (0.4%), 50-59 years old: *n*=32 (0.6%), 60-69 years old: *n*=39 (0.8%), 70-79 years old: *n*=35 (1.6%) for the TT-ET (no chemotherapy) group. In the < 30 years old category, < 10 patients were in the paclitaxel-TT (Tolaney) group.
(G) Combination of TT and systemic treatment regimen by age at BC diagnosis. The cohort is restricted to patients aged from 28 to 84 years (*n*=19,487).

Abbreviations: BC = breast cancer, TT = targeted therapy, ET = endocrine therapy.

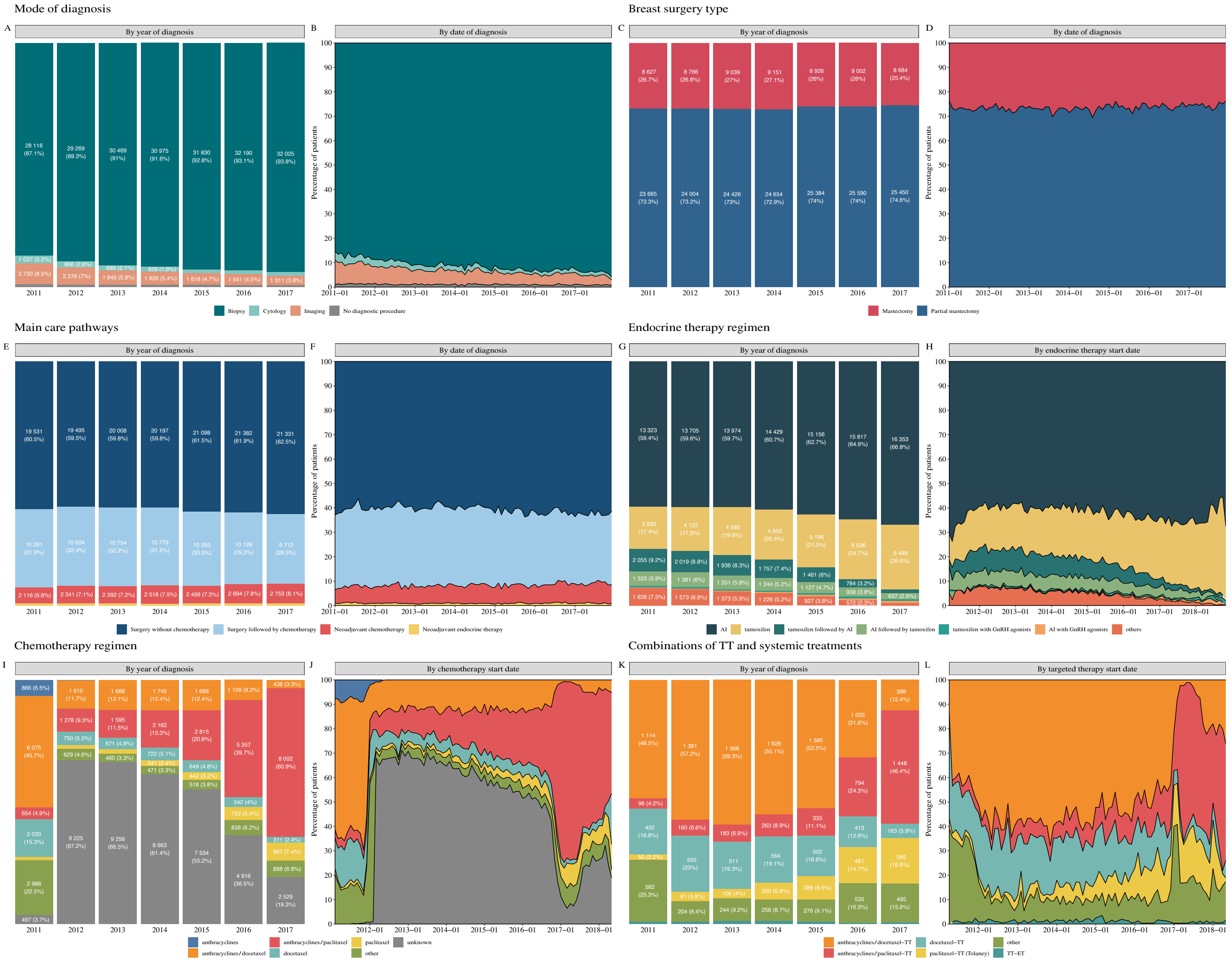

Figure S14: Changes in medical practices over time

(A) Mode of diagnosis by year of diagnosis. Raw data for subgroups representing less than 2% of the corresponding year class are not displayed on the graph, to ensure readability. The values by year class are: in 2014: *n*=659 (1.9%), 2015: *n*=514 (1.5%), 2016: *n*=553 (1.6%), 2017: *n*=490 (1.4%) for the cytology group. The values by year class for the no diagnostic procedure group are: in 2011: *n*=409 (1.3%), 2012: *n*=356 (1.1%), 2013: *n*=362 (1.1%), 2014: *n*=345 (1.0%), 2015: *n*=348 (1.0%), 2016: *n*=308 (0.9%), 2017: *n*=308 (0.9%);
(B) Mode of diagnosis by month-year of diagnosis (*n* = 235,368);
(C) Type of breast surgery by year of diagnosis;
(D) Type of breast surgery by month-year of diagnosis (*n* = 235,368);
(E) Main care pathways by year of diagnosis. Raw data for subgroups representing less than 2% of the corresponding year class are not displayed on the graph, to ensure readability. The values by year class are: in 2011: *n*=331 (1.0%), 2012: *n*=306 (0.9%), 2013: *n*=291 (0.9%), 2014: *n*=289 (0.9%), 2015: *n*=298 (0.9%), 2016: *n*=352 (1.0%), 2017: *n*=321 (0.9%) for the NET group;
(F) Main care pathways by month-year of diagnosis (*n*= 235,202). Patients who received neoadjuvant radiotherapy but not neoadjuvant chemotherapy are classified as NRT. They are not displayed on the plot (*n* =166).
(G) Endocrine therapy regimen by year of diagnosis. Raw data for subgroups representing less than 2% of the corresponding year class are not displayed on the graph, to ensure readability. The values by year class are: in 2011: *n*=31 (0.1%), 2012: *n*=42 (0.2%), 2013: *n*=52 (0.2%), 2014: *n*= 86 (0.4%), 2015: *n*=133 (0.6%), 2016: *n*=167 (0.7%), 2017: *n*=194 (0.8%) for the AI with GnRH agonists group. The values by year class are: in 2011: *n*=143 (0.6%), 2012: *n*=148 (0.6%), 2013: *n*=124 (0.5%), 2014: *n*=157 (0.5%), 2015: *n*=155 (0.6%), 2016: *n*=165 (0.7%), 2017: *n*=172 (0.7%) for the tamoxifen with GnRH agonists group. In the 2017 category, there were 338 (1.4%) patients in the tamoxifen followed by AI group and 271 (1.1%) patients in "others" group;
(H) Endocrine therapy regimen by month-year of ET start date. The cohort is restricted to patients whose month-year ET start date was after March 2011 and before November 2018 (*n* = 165,569);
(I) Chemotherapy regimen by year of diagnosis. Raw data for subgroups representing less than 2% of the corresponding year class are not displayed on the graph, to ensure readability: in the 2012 category, there were 16 (0.1%) patients in the anthracyclines group and 229 (1.7%) in the paclitaxel group; in the 2011 and 2013 categories, there were 194 (1.5%) and 244 (1.8%) patients, respectively, in the paclitaxel group;
(J) Chemotherapy regimen by month-year of CT start date. The cohort is restricted to patients whose month-year of CT start date was after February 2011 and before May 2018 (*n* = 95,175);
(K) Combinations of TT and other systemic treatments by year of diagnosis. Raw data for subgroups representing less than 2% of the corresponding year class are not displayed on the graph, to ensure readability. The values by year class are: in 2011: *n*=24 (1.0%), 2012: *n*=24 (1.0%), 2013: *n*=34 (1.3%), 2014: *n*=41 (1.4%), 2015: *n*=30 (1.0%), 2016: *n*=16 (0.5%), 2017: *n*=21 (0.7%) for the targeted therapy - endocrine therapy (no chemotherapy) group;

(L) Combinations of TT and other systemic treatments by month-year of TT start date. The cohort is restricted to patients whose month-year of TT start date was after April 2011 and before May 2018 (n = 19,658).
Abbreviations: BC = breast cancer, CT = chemotherapy, NAC = neoadjuvant chemotherapy; NET = neoadjuvant endocrine therapy, TT = targeted therapy, ET = endocrine therapy, AI = aromatase inhibitor.

Supplemental tables

Medical data	ICD-10
Carcinoma *in situ* of the breast	D05
Lobular carcinoma *in situ* of the breast	D050
Intraductal carcinoma *in situ* of the breast	D051
Other carcinoma *in situ* of the breast	D057
Unspecified type of carcinoma *in situ* of the breast	D059
Malignant breast tumor	C50
Malignant neoplasm of nipple and areola	C500
Malignant neoplasm of central portion of the breast	C501
Malignant neoplasm of upper-inner quadrant of the breast	C502
Malignant neoplasm of lower-inner quadrant of the breast	C503
Malignant neoplasm of upper-outer quadrant of the breast	C504
Malignant neoplasm of lower-outer quadrant of the breast	C505
Malignant neoplasm of axillary tail of the breast	C506
Malignant neoplasm of overlapping sites of the breast	C508
Malignant neoplasm of the breast of unspecified site	C509

Table S1: ICD-10 diagnosis codes used to identify breast cancer
Abbreviations : ICD 10 = International Statistical Classification and Related Health Problems – 10th revision.

	Medical Data	ICD-10	Medical Data	ICD-10
**Diagnosis code of other cancer**	Lip	C00	Ureter, bladder, other unspecified urinary organs	C66, C67, C68
	Tongue	C01, C02	Eye and adnexa, meninges	C69, C70
	Gum	C03	Brain	C71
	Floor of mouth	C04	Spinal cord, cranial nerves and other parts of CNS	C72
	Palate, other and unspecified parts of the mouth	C05, C06	Thyroid gland	C73
	Parotid and other salivary glands	C07, C08	Adrenal gland, endo glands and related structures, other ill-defined sites	C74, C75, C76
	Tonsil	C09		
	Oro/naso/hypopharynx	C10, C11, C13	Without specification of site	C80
	Pyriform sinus	C12	Hodgkin lymphoma	C81
	Lip, oral cavity and pharynx	C14	Follicular lymphoma	C82
	Esophagus, stomach	C15, C16	Non-follicular lymphoma	C83
	Small intestine, colon	C17, C18	Mature T/NK-cell lymphomas	C84
	Rectosigmoid junction, rectum	C19, C20	Other and unspecified types of non-Hodgkin lymphoma	C85
	Anus and anal canal	C21	Malig immunoproliferative dis and certain oth B-cell lymph	C88
	Liver and intrahepatic bile ducts	C22	Multiple myeloma and malignant plasma cell neoplasms	C90
	Gallbladder, other and unsp. parts of biliary tract	C23, C24	Lymphoid leukemia	C91
	Pancreas, other and ill-defined digestive organs	C25, C26	Myeloid leukemia	C92
	Nasal cavity and middle ear, accessory sinuses	C30, C31	Monocytic leukemia	C93
	Larynx, trachea, bronchus	C32, C33, C34	Other leukemias of specified cell type	C94
	Thymus, heart, mediastinum and pleura	C37, C38	Leukemia of unspecified cell type	C95
	Respiratory system and intrathoracic organs	C39	Other & unspecified malignant neoplasm of lymphoid, hematopoietic cells and tissues	C96
	Bone and articular cartilage of limbs or unsp. sites	C40, C41		
	Mesothelioma, Kaposi's sarcoma	C45, C46	Carcinoma *in situ* of oral, digestive, ear, respiratory, genital, unspecified organs	D00, D01, D02, D07, D09
	Peripheral nerves and autonomic nervous system	C47		
	Retroperitoneum and peritoneum	C48	Neoplasm of uncertain behavior of oral cavity and digestive organs	D37, D38, D39, D40, D41, D42, D43, D44
	Other connective and soft tissue	C49		
	Vulva, vagina	C51, C52		
	Corpus uteri, part unspecified	C54, C55	Myelodysplastic syndromes	D46
	Ovary, other and unsp. female genital organs	C56, C57	Other neoplasm of uncertain behavior of lymphoid, hematopoietic cells and tissues	D47
	Placenta	C58		
	Penis, other and unsp. male genital organs	C60, C63	Neoplasm of uncertain behavior of other and unsp sites	D48
	Kidney, renal pelvis	C64, C65		
	**Medical Data**	**ATC**	**Medical Data**	**ATC**
**Chemotherapy or immunotherapy molecules not indicated for breast cancer**	BENDAMUSTINE	L01AA09	RITUXIMAB	L01XC02
	BUSULFAN	L01AB01	CETUXIMAB	L01XC06
	CARMUSTINE	L01AD01	PANITUMUMAB	L01XC08
	FOTEMUSTINE	L01AD05	IPILIMUMAB	L01XC11
	RALTITREXED	L01BA03	TEMSIROLIMUS	L01XE09
	PEMETREXED	L01BA04	TOPOTECAN	L01XX17
	CLADRIBINE	L01BB04	IRINOTECAN	L01XX19
	CLOFARABINE	L01BB06	ARSENIC TRIOXIDE	L01XX27
	CYTARABINE	L01BC01	BORTEZOMIB	L01XX32
	AZACITIDINE	L01BC07	ALEMTUZUMAB	L04AA34
	TRABECTEDIN	L01CX01	LENALIDOMIDE	L04AX04
	IDARUBICIN	L01DB06		

Table S2: ICD-10 and ATC codes used to identify cancer at another site.
Abbreviations: ICD-10 = International Statistical Classification and Related Health Problems – 10th revision ; ATC = Anatomical Therapeutic and Chemical

		Medical data	ICD-10	ATC
**Diagnosis code for metastatic disease**		Metastatic disease	C770, C771, C772, C774, C775, C778, C779, C78, C780, C781, C782, C783, C784, C785, C786, C787, C788, C79, C790, C791, C792, C793, C794, C795, C796, C797, C798, C799	
**Molecules indicated only for metastatic disease**	**Chemotherapy**	CAPECITABINE		L01BC06
		CARBOPLATIN		L01XA02
		CISPLATIN		L01XA01
		DOXORUBICIN (only CAELYX®)		L01DB01
		ERIBULIN		L01XX41
		ETOPOSIDE		L01CB01
		GEMCITABINE		L01BC05
		MELPHALAN		L01AA03
		METHOTREXATE		L01BA01
		MILTEFOSINE		L01XX09
		MITOMYCIN		L01DC03
		MITOXANTRONE		L01DB07
		PIRARUBICIN		L01DB08
		THIOTEPA		L01AC01
		VINBLASTINE		L01CA01
		VINCRISTINE		L01CA02
		VINDESINE		L01CA03
		VINORELBINE		L01CA04
	***HER2*- Targeted Therapy**	LAPATINIB		L01XE07
	**Non-*HER2* Targeted Therapy**	BEVACIZUMAB		L01XC07
		BYL719 (ALPELISIB)		L01XE
		EVEROLIMUS		L01XE10
		PALBOCICLIB		L01XE33
	**Endocrine Therapy**	TOREMIFENE		L02BA02
		FORMESTANE		L02BG02
		FULVESTRANT		L02BA03
	**Other**	DEXRAZOXANE		V03AF02
		SAMARIUM (153SM) LEXIDRONAM		V10BX02

Table S3: ICD-10 and ATC codes used to identify *de novo* metastatic breast cancer.

Abbreviations: ICD 10 = International Statistical Classification and Related Health Problems – 10th revision; ATC = Anatomical Therapeutic and Chemical Classification.

		Medical Data	CCAM	ICD-10	ATC
Surgery		Mastectomy with axillary surgery	QEFA003, QEF005, QEFA010, QEFA020		
		Mastectomy without axillary surgery	QEFA007, QEFA012, QEFA013, QEFA015, QEFA019		
		Partial mastectomy with axillary surgery	QEFA001, QEFA008		
		Partial mastectomy without axillary surgery	QEFA004, QEFA016, QEFA017, QEFA018		
		Axillary surgery without breast surgery	FCFA021, FCFA029		
Radiotherapy		Appointment for antineoplastic radiation therapy		Z5100	
		Radiotherapy preparation session		Z5101	
		Radiotherapy session		Z510	
		Radiotherapy procedures	ZZNA002, ZZNL001, ZZNL002, ZZNL003, ZZNL004, ZZNL005, ZZNL006, ZZNL009, ZZNL011, ZZNL012, ZZNL013, ZZNL014, ZZNL015, ZZNL016, ZZNL017, ZZNL018, ZZNL019, ZZNL02, ZZNL030, ZZNL031, ZZNL036, ZZNL037, ZZNL039, ZZNL040, ZZNL042, ZZNL043, ZZNL045, ZZNL046, ZZNL048, ZZNL049, ZZNL050, ZZNL051, ZZNL052, ZZNL053, ZZNL054, ZZNL055, ZZNL058, ZZNL059, ZZNL060, ZZNL061, ZZNL062, ZZNL063, ZZNL064, ZZNL065, ZZNL900, ZZNL902, ZZNL903, ZZNL904, ZZNL905, ZZNL906, YYYY016, YYYY021, YYYY023, YYYY045, YYYY046, YYYY047, YYYY048, YYYY049, YYYY050, YYYY051, YYYY052, YYYY053, YYYY054, YYYY055, YYYY056, YYYY080, YYYY081, YYYY099, YYYY101, YYYY109, YYYY122, YYYY128, YYYY136, YYYY141, YYYY151, YYYY152, YYYY166, YYYY175, YYYY197, YYYY1211, YYYY223, YYYY225, YYYY244, YYYY256, YYYY267, YYYY299, YYYY301, YYYY302, YYYY303, YYYY304, YYYY305, YYYY306, YYYY307, YYYY310, YYYY312, YYYY313, YYYY314, YYYY315, YYYY316, YYYY320, YYYY323, YYYY324, YYYY325, YYYY326, YYYY327, YYYY331, YYYY334, YYYY335, YYYY336, YYYY337, YYYY338, YYYY343, YYYY345, YYYY346, YYYY347, YYYY348, YYYY349, YYYY356, YYYY357, YYYY358, YYYY359, YYYY360, YYYY365, YYYY367, YYYY368, YYYY369, YYYY370, YYYY371, YYYY377, YYYY379, YYYY380, YYYY381, YYYY382, YYYY383, YYYY387, YYYY390, YYYY391, YYYY392, YYYY393, YYYY398, YYYY450, YYYY451, YYYY457, YYYY458, YYYY459, YYYY460, YYYY468, YYYY469, YYYY470, YYYY471, YYYY479, YYYY480, YYYY481, YYYY491, YYYY492, YYYY493, YYYY497, YYYY500, YYYY511, YYYY520, YYYY522, YYYY533, YYYY544, YYYY555, YYYY566, YYYY577, YYYY588, YYYY599		
Chemotherapy		Encounter for antineoplastic chemotherapy and immunotherapy		Z511, Z512	
		Anticancerous agent administration	ZZLF900		
		Port implanting	EBLA001		
		Cerebral anticancerous agent administration	ABLB006		
		Intrathecal anticancerous agent administration	AFLB003, AFLB013		
		Upper limb arterial anticancerous agent administration	ECLF005, ECLF006		
		Lower limb arterial anticancerous agent administration	EELF004, EELF005		
		Liver anticancerous agent *in situ* administration	EDLF014, EDLF015, EDLF016, EDLF017		
		Kidney anticancerous agent *in situ* administration	EDLF018, EDLF019, EDLF020, EDLF021		
		Cervical/cephalic anticancerous agent administration	EBLF002, EBLF003		
		Intrapleural anticancerous agent administration	GGLB001, GGLB008		
		Intraperitoneal anticancerous agent administration	HPLB002, HPLB003, HPLB007		
		Locoregional anticancerous agent administration with extracorporeal circulation	ZZLF004		
		Locoregional anticancerous agent administration without extracorporeal circulation	ZZLF900		
		CYCLOPHOSPHAMIDE			L01AA01
		DOCETAXEL (identifiable until March 2012)			L01CD02
		EPIRUBICIN			L01DB03
		FLUOROURACIL			L01BC02
		PACLITAXEL			L01CD01
Targeted Therapy	*Anti HER2*	PERTUZUMAB			L01XC13
		TRASTUZUMAB			L01XC03, L01XC14
Endocrine Therapy	Tamoxifen	TAMOXIFEN			L02BA01
	Aromatase Inhibitor	ANASTROZOLE			L02BG03
		EXEMESTANE			L02BG06
		LETROZOLE			L02BG04
	GnRH agonists	GOSERELIN			L02AE03
		LEUPRORELIN			L02AE02
		TRIPTORELIN			L02AE04

Table S4: CCAM, ICD-10 and ATC codes used to identify breast cancer treatments.

Abbreviations: CCAM = French Common Classification of Medical Procedures; ICD 10 = International Statistical Classification and Related Health Problems – 10th revision; ATC = Anatomical Therapeutic and Chemical Classification; HER2=human epidermal growth factor receptor 2.

Medical data	CCAM	ATC	CIP	UCD
Celioscopic oocyte extraction	JJFC011			
Vaginal oocyte extraction	JJFJ001			
Chorionic gonadotrophin		G03GA01		
Choriogonadotropin alfa		G03GA08		
Short-acting gonadotrophin agonists			3285026, 3285032, 3287924, 3285061, 3285055, 3328563, 3535210	9109419, 9112077, 9112060, 9148603, 9219958
Gonadotrophin antagonist			3717790, 3517815, 3553018, 3553024	9218812, 9218829, 9233591

Table S5: CCAM, ATC, CIP and UCD codes used to identify embryo or oocyte cryopreservation

Abbreviations: CCAM = French Common Classification of Medical Procedures; ATC = Anatomical Therapeutic and Chemical Classification; CIP (*Code Identifiant de Présentation*); UCD (*Unités Communes de Dispensation*)

Medical data		CCAM
**Breast core biopsy**		QEHA001, QEHA002, QEHB002, QEHJ001, QEHH001, QEHJ005, QEHJ006, QEHH002, QEHH015, QEHJ004
**Fine needle aspiration cytology**		QEHB001, QEHJ002, QEHJ003, QEHH003, QEHP002
**Breast imaging procedures**	Mammography	QEQK001, QEQK004, QEQK005
	Mammary ultrasound	QEQM001
	Mammary MRI	QEQJ001, QEQN001
	CT scan	QEQH002, QEQK006
	Galactography	QEQH001

Table S6: CCAM codes used to identify diagnostic procedures for breast cancer

Abbreviations: CCAM = French Common Classification of Medical Procedures; CT = computed tomography.
